# Exosomes and Exosomal miRNA in Respiratory Diseases

**DOI:** 10.1155/2016/5628404

**Published:** 2016-09-25

**Authors:** Shamila D. Alipoor, Esmaeil Mortaz, Johan Garssen, Masoud Movassaghi, Mehdi Mirsaeidi, Ian M. Adcock

**Affiliations:** ^1^Clinical Tuberculosis and Epidemiology Research Center, National Research Institute of Tuberculosis and Lung Diseases (NRITLD), Shahid Beheshti University of Medical Sciences, Tehran, Iran; ^2^Institute of Medical Biotechnology, Molecular Medicine Department, National Institute of Genetic Engineering and Biotechnology (NIGEB), Tehran, Iran; ^3^Division of Pharmacology, Utrecht Institute for Pharmaceutical Sciences, Faculty of Science, Utrecht University, Utrecht, Netherlands; ^4^Airways Disease Section, National Heart & Lung Institute, Imperial College London, London, UK; ^5^Nutricia Research Centre for Specialized Nutrition, Utrecht, Netherlands; ^6^Department of Pathology and Laboratory Medicine, University of California, Los Angeles (UCLA), Los Angeles, CA, USA; ^7^Division of Pulmonary, Critical Care, Sleep and Allergy, Department of Medicine, University of Miami Leonard M. Miller School of Medicine, Miami, FL, USA

## Abstract

Exosomes are nanosized vesicles released from every cell in the body including those in the respiratory tract and lungs. They are found in most body fluids and contain a number of different biomolecules including proteins, lipids, and both mRNA and noncoding RNAs. Since they can release their contents, particularly miRNAs, to both neighboring and distal cells, they are considered important in cell-cell communication. Recent evidence has shown their possible importance in the pathogenesis of several pulmonary diseases. The differential expression of exosomes and of exosomal miRNAs in disease has driven their promise as biomarkers of disease enabling noninvasive clinical diagnosis in addition to their use as therapeutic tools. In this review, we summarize recent advances in this area as applicable to pulmonary diseases.

## 1. Introduction

Biological markers (biomarkers) were initially described by Hulka as “cellular, biochemical or molecular alterations that are measurable in biological media such as human tissues, cells or fluids” [1]. Today this definition has been broadened and includes biological features that can be measured and evaluated to reflect a particular physiological or biological state and includes specific analytes through to physiological measures such as blood pressure. Biomarkers act as indicators to enable the evaluation of a normal or pathogenic condition or the response to therapy [2].

In pulmonary diseases, biomarkers are powerful tools in understanding the spectrum of pathological conditions affecting the lung microenvironment and function as well as predicting drug responses. The expressions of many proteins, lipids, and genomic biomarkers have been investigated due to their possible central roles in the biology of human lung diseases [3]. MicroRNAs (miRNAs) have been studied in many diseases due to their utility in disease diagnostics for monitoring therapy and to predict the probability of disease recurrence [4]. Recent studies have shown that miRNAs have central roles in multiple aspects of lung inflammation and disease pathogenesis [3–9].

Exosomes are extracellular membrane vesicles involved in cell-cell communication by shuttling various molecules including miRNAs from donor to recipient cells [10]. Exosomal miRNAs delivered to target cells can significantly affect biological pathways within target/recipient cells resulting in altered cellular function and the development of a pathological state [11]. The packaging of miRNAs within the exosomal lipid bilayers protects them from enzymic degradation by body fluids resulting in a relatively long and stable duration of expression [11, 12]. In this review, we aim to summarize recent advances regarding the status of exosomes and exosomal miRNAs as potential biomarkers in lung diseases.

## 2. Exosomes Properties and Function

**Figure 1:**
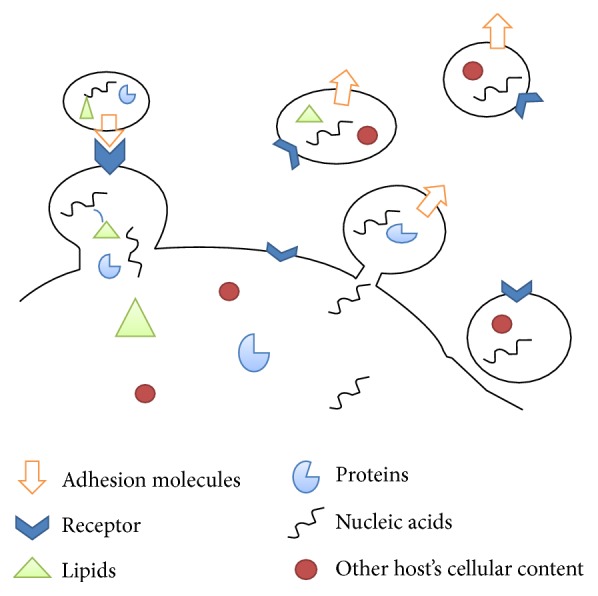
Exosomes properties and function exosomes are secreted membrane vesicles released into the extracellular space and transfer proteins, lipids, nucleic acids, and other hosts' cellular content. The fusion of the exosome membrane with the target cell plasma membrane results in the release of exosome content into the target cell cytoplasm.

Exosomes are small, 30–120 nm in diameter, cell-derived vesicles which are secreted from most cell types (Figure 1). They are ubiquitous in body fluids including urine, plasma, breast milk, bronchoalveolar lavage (BAL) fluid, saliva, seminal fluid, amniotic liquid, ascites, synovial fluid, breast milk, and cerebrospinal fluid (CSF) [13, 14]. The initial discovery of exosomes in the mid-1980s referred to small vesicles that bud from reticulocytes during their maturation to eliminate some membrane bound proteins [15]. These exosomes were originally thought to function as the cell's “garbage bin” and so received little attention but further studies indicated that these small exocytosed vesicles were not specific to reticulocytes and were released from most mammalian cells [14]. Later, exosomes were shown to have immune regulatory effects, with the demonstration that B-cell-derived exosomes could stimulate T cells [16, 17]. Additional studies highlighted the role of exosomes in tumorigenicity, immune modulatory processes, neurodegenerative disease, and the transfer of infectious agents [18–20]. The turning point in exosome research was in 2007 with the finding that exosomes released from mast cells contain over 1200 mRNAs which could be transferred to other cells and be translated into proteins [21, 22].

**Figure 2:**
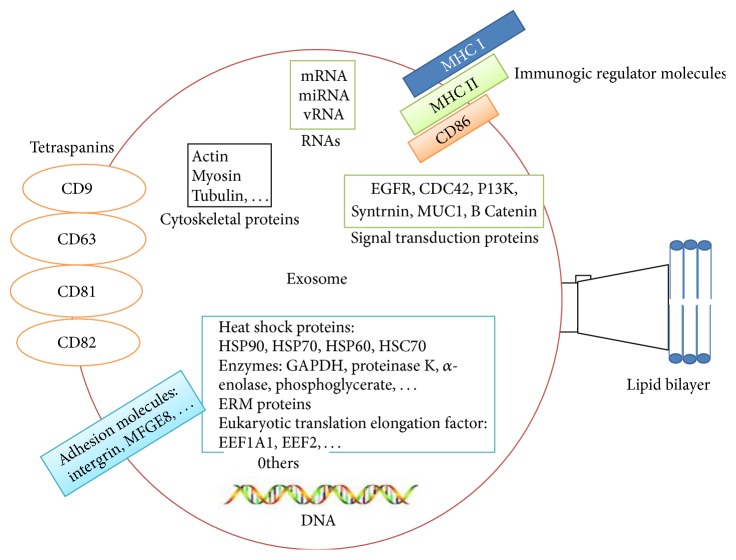
Structure and contents of exosomes: exosomes contain a plasma membrane-derived phospholipid bilayer membrane. Exosomal contents based on the cell type of origin include mRNA, miRNA, and DNA and proteins such as annexins, tetraspanins, MHC molecules, cytoskeletal proteins, enzymes, and signal transduction proteins.

According to ExoCarta, a database of exosomal proteins, RNA, and lipids [23], exosomes have a complex composition whose molecular content is dependent upon their cell of origin. 4,563 proteins, 194 lipids, 1,639 mRNAs, and 764 miRNAs have been recognized in exosomes from different species with membrane transport proteins and fusion proteins being most frequently detected [23]. Some exosomal proteins are universal including tetraspanins, CD63, CD81, CD9, and heat shock protein (Hsp70) and these are commonly used as exosomal markers [24]. Exosomes are rich in lipids such as cholesterol, phospholipids, phosphatidylserine, and prostaglandins but lack nuclear, mitochondrial, and ribosomal proteins but, importantly in the context of this review, contain both mRNAs and miRNAs [23] (Figure 2).

Circulating exosomes are highly stable in biological fluids [11] and can, therefore, provide a great deal of information about the physiological and pathological status of the originating cell via accessing their molecular contents [15, 25, 26]. As a result, comprehensive analysis of exosomal miRNA is defining novel therapeutic and diagnostic targets for a variety of pulmonary diseases [26].

## 3. The Potential of Exosomes in Diagnostics

Exosomes can pass through the blood-brain barrier and move to distant tissues, where they fuse with the cell membranes of target cells to transfer their contents [24]. Due to their donor cell characteristics and the ability to provide cell-cell communication, exosomes are increasingly viewed as disease biomarkers which require minimally invasive procedures to interrogate the biology of difficult-to-access cells and organs [27].

The presence of pathogen-derived antigens in the exosomes was firstly suggested by Beatty et al. in 2000 [28]. Exosomes released by infected cells carry a variety of pathogen-derived molecules which can act as biomarkers for specific infectious agents [16, 29] exemplifying the potential role of exosomes in the diagnosis of infectious diseases. In allergic mouse models of asthma, exosomes obtained from bronchoalveolar lavage (BAL) of allergic mice protect naïve mice against airway inflammation [30].

The number and contents of exosomes in body fluids may change significantly with disease. For example, there are increased numbers of BAL exosomes in sarcoidosis patients compared with healthy volunteers [31]. BAL exosomes from sarcoidosis patients induce the production of inflammatory cytokines by PBMCs and promote the release of CXCL-8 by airway epithelial cells through delivery of pathogen-associated proinflammatory mediators [31]. In addition, increased numbers of circulating exosomes are associated with disease progression in cancer [30, 32]. The exosomal content may also provide valuable information about disease status [33]. Exosomes from macrophages from patients with active* M. tuberculosis* infection contain a highly antigenic mycobacterium protein compared with the lack of antigenic protein observed in patients with latent infection [34, 35]. In addition, exosomes derived from* Mycobacterium avium* infected macrophages also contain pathogenic proinflammatory glycopeptidolipids [16].

Different exosomal content signatures are also found in bladder and gastric cancer [36, 37]. 8 proteins are differentially expressed in urinary exosomes of patients with bladder cancer and these are indicated as potential disease biomarkers [38]. Enhanced expression of oncogenic HER-2/neu and MAGE-1 mRNA is found in exosomes isolated from various body fluids of patients with gastric cancer [38].

Overall, the analysis of exosomal content may be useful for the early detection of various diseases including cancers and infectious diseases and a sensitive diagnostic test for prostate cancer based on analysis of exosomal proteins was recently launched (http://www.carislifesciences.com/) [39].

## 4. The Potential of Exosomes in Therapeutics

Exosomes are important regulators of many functions including tissue homeostasis, immune stimulatory or immunosuppressive functions, and tumorigenicity [30]. In addition, exosomes released from antigen presenting cells (APCs) contain MHC-I, MHC-II, and CD86 and can act in a similar manner to APCs [13, 17, 40, 41] and dendritic cell- (DC-) derived exosomes are as effective as DCs in inducing some immune responses [42]. For example, exosomes derived from DCs pulsed with tumor antigens induce a very strong immune response resulting in the rejection of established tumors in mice [30]. This phenomenon probably occurs due to the high density of tumor antigens and the presence of HSPs in exosomes which act like an adjuvant. In addition, exosomes containing HSP70 have recently been shown to be proinflammatory by activating natural killer cells (NKs) and macrophages [43].

These properties of exosomes have been utilized in phase I [44, 45] and phase II clinical trials for inoperable non-small cell lung cancer. NK cell-derived exosomes, containing perforin and granzyme B, have also shown antitumor activities in vitro or in vivo [43, 46]. Further, it was observed that exosomes derived from tumor cells (tumor exosomes or TEX) can induce an antitumor immune response [30, 47]. TEX suppress NK cell function via modulation of NKG2D receptor expression [43, 48]. It was later shown that immunization of mice with TEX or with DCs pulsed with TEX led to a significant reduction in tumor growth and an increased survival [49, 50] and the use of exosomes in cancer immunotherapy is promising.

However, exosomes have also been reported to be involved in promoting cancer growth. For example, exosome-mediated transportation of the oncoproteins K-RAS and MET and their uptake by other cells promote an optimal local tumor microenvironment [51]. TEX may contain different levels of oncogenic miRNAs which may affect acceptor cell function via posttranscriptional modulation [25]. Metastasis requires a specific microenvironment termed “premetastatic niche,” which allows the colonization and growth of tumor cells in a secondary organ at unique distant sites. The formation of premetastatic niches has been attributed to exosomes directing the disseminated tumor cells to future metastatic sites [52, 53]. Exosome-mediated metastasis occurs in a nonrandom manner and is directed to the future site of metastasis according to the TEX integrin expression profile [54].

The potential role of exosomes in the treatment of allergy and infectious diseases has also been investigated. Exosomes released from DCs laden with pathogen-derived antigens can protect against infection [30]. In contrast, exosomes can also contain exogenous viral RNAs and be involved in the spreading of infection [55]. On the other hand, due to their biological properties, exosomes have also been proposed as possible delivery vectors for therapeutic purposes [56] and vaccine delivery vehicles [57, 58]. Indeed, the first two phase I trials in human cancer have been published using exosomes from monocyte-derived DCs loaded with tumor antigens [44, 59–61].

## 5. Putative Roles of Exosomes in Lung Microenvironment and Pathogenesis

The lung is a unique organ considering the broad range of cells that are found within the parenchyma and airway structures. Cell-cell communication is essential for the optimal functioning of the lung and so exosomes are expected to be important players in lung biology and function [12]. Exosomes are released by a wide range of cell types present within the lung including endothelial cells, stem cells, epithelial cells, alveolar macrophage, and tumor cells, although epithelial cells are reported to be the main source of lung-derived exosomes [62]. Exosomes released by airway epithelial cells contain mucins and alpha 2,6-linked sialic acid which have a neutralizing effect on human influenza virus infection [12]. Membrane-tethered mucins within epithelial cell-derived exosomes affect the structural properties, conformation, and surface charge of exosomes. The properties of exosomes contribute to mucociliary defense by the lung's innate immune system [63, 64].

Exosomes control inflammatory signaling within the airway through intercellular communication [4] as elegantly demonstrated by the transfer of suppressor of cytokine signaling (SOCS)1 from macrophage-derived exosomes to alveolar epithelial cells. This attenuates the activation of signal transducer and activator of transcription (STAT) both in vitro and in vivo [62]. Exosomes derived from alveolar macrophages also regulate airway inflammation through the transfer of miR-223 to various respiratory cells resulting in cellular homeostasis and differentiation [65].

Exosomes may also act as a part of the stress response in the airway. In sarcoidosis, exosomes cause the initiation and progression of inflammatory responses by enhancing the induction of IL-13, INF-gamma, and CXCL-8 production in the lung microenvironment [31]. Furthermore, the secretion of exosomes and the composition of the secreted exosomes can be altered following infection. For example, it was shown that alveolar macrophage-derived exosomes are enriched for HSP-70 after infection with* Mycobacterium* [66].

## 6. Biomarker Discovery Using Exosomal miRNAs in Lung Diseases

Based on the available evidence, various types of cell-derived exosomes may regulate airway homeostasis and contribute to the pathogenesis of various lung diseases [62]. One of the most attractive aspects of exosome research in pulmonary disease is in the context of novel diagnostic and/or prognostic biomarkers for the early detection and improved treatment of patients particularly with respect to lung cancer. The miRNA profiles of TEX are similar to the corresponding tumor miRNA signatures [67] and are also related to their donor cell inflammatory status [25, 68, 69]. This suggests that exosomal miRNAs may act as potential noninvasive biomarkers for the diagnosis of lung cancer and other pulmonary diseases [12, 70].

The crucial roles of exosomes in the initiation and development of inflammatory airway diseases such as asthma, COPD, sarcoidosis, and tuberculosis and their possible role as biomarkers are reviewed further in the following section.

## 7. Tuberculosis

Eradication of tuberculosis (TB) has been hampered partly by the ability of* Mycobacterium* tuberculosis (MTB) to remain dormant in the human body for years without causing disease, a state referred to as latent tuberculosis [71]. Comprehensive proteomic based analysis has determined the protein content of exosomes derived from macrophages infected with either live or dead* M. tuberculosis* in vitro [72]. This revealed the dominant presence of host proteins along with 41 mycobacterial proteins within the secreted exosomes. Further analysis indicated the presence of highly immunogenic* Mycobacterium* proteins including antigen SAT-6 (Rv3875), Ag85 complex (Rv3804c, Rv1886c, and Rv0129c), MPT64 (1980c), and MPT63 (1926c) [7, 57, 58]. Subsequent studies identified twenty mycobacterial proteins in exosomes isolated from the serum of TB patients including the antigens 85b, BfrB, GlcB, and Mpt64 [34].

In addition, it is possible to distinguish pulmonary and extrapulmonary TB based on exosomal markers in serum such as MPT64 [34] and to recognize active and latent disease [34]. The ability to detect latent from active infection is particularly important within an endemic population and could improve monitoring of at risk persons and prevent the transmission of infection. Importantly, some of the M. tuberculosis products found in exosomes were identical in cell culture, animal models, and human clinical specimens [8, 57].

Exosomes released from CFP-treated macrophages (CFP:* M. tuberculosis* culture filtrate proteins) are able to activate both innate and acquired immune responses. In these exosomes, 29* M. tuberculosis* proteins were detected with the majority overlapping with those present in the exosomes isolated from* M. tuberculosis*-infected macrophages. These exosomes could stimulate macrophages, DCs, and naïve T cells in vivo. This indicates that exosomes with* M. tuberculosis* antigen cargo may be a suitable vehicle for the development of tuberculosis CFP-based vaccines [7].

Exosomes are also reported to act as carriers of pathogen-associated molecular patterns (PAMPs) and to affect recipient cells by either silencing or promoting the immune responses [70].* M. tuberculosis (Mtb)* can induce partial resistance to INF-gamma stimulation in infected macrophages via PAMPs such as the 19 kDa lipoprotein and mycolyl-arabinogalactan-peptidoglycan complex (mAGP complex) binding to Toll-like receptor (TLR)2 on macrophages [73]. This effect is mimicked by exosomes released from* Mtb*-infected macrophages [20].

Interestingly, it has been shown that genome-wide* Mtb* infection induced miRNA expression profile in primary human macrophages [74]. In this way infection of human macrophages with virulent* Mtb* H37Rv and avirulent* M. bovis* BCG results in a pattern of miRNA expression mostly overlapping between the two live mycobacteria considered, while a substantially different pattern emerged from infection with killed* Mtb* bacilli suggesting an active influence of live intracellular bacteria on cell target miRNA metabolism [74].

Overall, the amount of exosomal miRNAs from* M. tuberculosis*-infected macrophages is significantly lower in comparison with those from uninfected cells. Additionally, more than 100 mRNAs were unique to the exosomes from the infected cells and these may be involved in the regulation of immune responses in recipient cells [75]. This data supports the functional and diagnostic potential of exosomal mRNAs and miRNAs in tuberculosis.

## 8. COPD

The persistence of inflammation is characteristic of COPD [76] and it is plausible that exosomes play a key role in COPD by regulating this inflammation [77]. COPD is induced by chronic exposure of the airway to irritants including cigarette smoke which leads to epithelial cell injury, destruction of pulmonary capillary vasculature, acceleration of epithelial cell senescence, and airway remodeling which results in the loss of lung function [78, 79]. Endothelial cell injury within the lung parenchyma is an important factor in emphysema [80] and a group of endothelial-derived microparticles (EMPs) are increased in patients with stable COPD and during exacerbation. These EMPs contain vascular endothelial-cadherin, platelet endothelial cell adhesion molecule, and E-selectin. Importantly, the level of EMPs in stable COPD significantly correlated with lung destruction and airflow limitation [81]. These results indicate the close relationship between endothelial lung injury and EMP function in the pathophysiology of COPD.

Airway epithelial cell injury is also important in COPD pathogenesis. Injured lung epithelial cells are a source of inflammatory mediators such as TNF-*α*, IL-1*β*, GM-CSF, TGF*β*, and CXCL-8 which may act in both autocrine and paracrine manners. TGF*β* induces the remodeling of airway cells by regulation and promoting of myofibroblast differentiation which is the main cause of fibrosis development during airway remodeling. The level of TGF*β* in the small airway epithelium of COPD patients is correlated with the severity of airway obstruction [8].

The paracrine activity of these mediators is mediated, at least in part, by exosomes [82]. This mechanism has been suggested as causing epithelial cell death and lung tissue loss upon chronic exposure of cigarette smoke (CS). Prolonged exposure to CS induced the release of the CCN1-enriched exosomes from lung epithelial cells. CCN1 plays an important role in tissue remodeling and repair process as an extracellular matrix protein [83] and enhances CXCL-8 release from cells via the Wnt signaling pathway [84]. Hence, CCN1-enriched exosomes could lead to the paracrine induction of CXCL-8 secretion in the lung mesenchyme or parenchyma and the subsequent recruitment of inflammatory cells such as neutrophils which will result in lung tissue fibrosis [62, 83, 85]. CS can increase the numbers of circulating lung epithelial cell-derived exosomes [86] and the degree of lung endothelial injury in COPD may be predicted by circulating exosomes [2]. This suggests that, in addition to being important drivers of COPD pathophysiology, exosomes may also serve as biomarkers of COPD progress and treatment [81].

Alpha 1 anti-trypsin (AAT1) deficiency is observed in ~1.5% of COPD patients [81, 86]. AAT1 is a glycoprotein serine protease which protects the lung from inflammatory insults [87]. Lung endothelial cells transport AAT1 to the alveolar epithelium and air spaces packaged in exosomes which are rapidly internalized by epithelial cells. Efficient trafficking of AAT1-containing exosomes across an uninjured lung endothelial barrier may be involved in the protective effect against CS [88].

Epithelial mesenchymal transition (EMT) is another important component of small airway remodeling and fibrosis in COPD [89, 90]. Increased urokinase plasminogen activator receptor (uPAR) expression in the small airway epithelium is a sign of an active EMT process occurring in patients with COPD [87]. EMT can also be linked with angiogenesis (termed EMT-type-3) in the large airway resulting in the formation of a procancer stromal niche [91–94]. Up to 70% of lung cancers occur in COPD patients with mild-to-moderate disease [95]. Based on the ability of exosomes to transfer bioactive molecules between cells and to affect recipient cell function, a role for exosomes in EMT has been proposed [91]. Disruption of the epithelial mesenchymal trophic unit (EMTU) is observed in COPD [94]. The EMTU consists of one epithelial cell layer, the basement membrane zone (BMZ), and mesenchymal cells including fibroblasts [96]. EMTU fibroblasts are involved in repair and remodeling processes associated with the increased small airway thickness in COPD [97] possibly as a result of extensive transfer of paracrine mediators by exosomes [62].

The plasma level of circulating muscle-specific miRNAs (myomirs) is different between COPD and non-COPD subjects and there is an association between the reduction of some myomirs and skeletal muscle weakness and changes in quadriceps fiber composition [98]. It is suggested that the plasma level of exosomal miRNA can reflect changes within the skeletal muscle and so can act as a biomarker of skeletal muscle dysfunction [98]. A recent abstract has reported on the level of exosomal miRNAs in BAL fluid and serum of mild-to-moderate COPD patients compared with healthy controls [99]. One miRNA was significantly upregulated in serum and 4 miRNAs were significantly downregulated in BAL fluid of COPD patients. In silico and in vitro analysis identified ribosomal s6 kinase (S6K) as the main target of these miRNAs. S6K is part of the mTORC1 signaling pathway which is a key regulator of skeletal muscle wasting. This highlights the potential of exosomal miRNAs as noninvasive diagnostic biomarkers [99].

## 9. Sarcoidosis

Pulmonary sarcoidosis is a systematic, inflammatory disease with unknown etiology. It is characterized by the formation of noncaseating granulomas predominantly in lung and the presence of interferon- (IFN-) producing T cells which leads to inflammation and tissue damage in multiple organs, especially the lung [31, 100]. In the context of the molecular pathogenicity of sarcoidosis, the expression profile of intracellular miRNAs has been reported in a number of studies [101–103] but there is limited information regarding exosomes and exosomal miRNAs in sarcoidosis. The first study demonstrated that increased numbers of exosomes could be isolated from the BAL fluid of sarcoidosis patients compared to healthy individuals. The BAL exosomes from sarcoidosis patients induced higher levels of IFN*γ* and interleukin-13 production by PBMCs and CXCL-8 production by epithelial cells in comparison to that induced by exosomes from healthy individuals [31].

In a recent abstract, the profile of exosomal miRNA in BAL fluid and serum of sarcoidosis patients has been investigated. In this study, miR-21, miR-26a, and miR-146a miRNAs were detected in BAL exosomes by RT-PCR with no expression of miR-15a, miR-129, miR-133a, miR-133b, miR-134, miR-195, miR-452, and miR-589 observed [104]. This study indicated the potential roles of exosomes in the initiation and progression of inflammation in sarcoidosis but more work is needed to fulfill the promise that exosomes may be a new potential target for the clinical treatment of sarcoidosis.

## 10. Asthma

Asthma is a heterogeneous chronic inflammatory airways disease, characterized by reversible airway narrowing and/or airway hyperresponsiveness in response to nonspecific stimuli such as exercise, allergens, infections, and air pollutants [105]. The main features of asthma are due to the pathophysiological effects of proinflammatory cytokines such as IL-4, IL-5, and IL-13 released by activated CD4^+^ T cells in response to environmental stimuli [105]. Expression of these mediators results in increased numbers or activation status of mast cells, Th2 cells, and eosinophils along with airway remodeling, reversible airway hyperresponsiveness, and airway obstruction [106].

Exosomes released from the key cells involved in asthma such as mast cells, eosinophils, DCs, T cells, and bronchial epithelial cells can induce priming and activation of other asthma-associated cells. For example, DC-derived exosomes activate allergen-specific Th2 cells [107]. These exosomes contain costimulatory molecules and major histocompatibility complex classes I and II on their surfaces which help them to induce the antigen-specific activation of T cells [108]. In addition, eosinophil-derived exosomes are increased in asthmatic patients and can modulate features of asthma in vitro after transfer to recipient cells [109].

Exosomes may also have beneficial effects. Thus, despite exosomes being able to enhance allergic responses, tolerizing exosomes that block allergic responses and prevent the initiation and development of an allergic response have also been reported [110]. Intranasal transfer of BAL fluid-derived exosomes from a tolerized mouse prevents allergic sensitization. It has been suggested that exosomes-based vaccines could be a therapeutic option for asthma and other allergic diseases [110].

The exosome profiles of BAL fluid of asthmatic patients were compared to those from healthy individuals. The BAL-derived exosomes from patients with asthma promoted production of CXCL-8 and leukotriene C4 by bronchial epithelial cells. These BAL-derived exosomes contain the epithelial marker mucin 1 on their surface suggesting that they originated from bronchial epithelial cells [111].

IL-13 can drive airway epithelial cells to produce exosomes which, in turn, promoted the proliferation of undifferentiated lung macrophages [112]. The profile of BAL-derived exosomal miRNAs in asthma shows differential expression of 24 miRNAs compared to those from healthy individuals. A number of these altered miRNAs are involved in IL-13-mediated events and a significant correlation was seen between the expression of these miRNAs and pulmonary function [113]. Further studies on exosomes and exosomal miRNAs in asthma will provide additional insight into cell-cell communication in asthma and may also help define the mechanism(s) underlying the various subgroups of patients with this disease.

## 11. Other Respiratory Diseases

In addition to the above-mentioned respiratory diseases, the role of exosomes in some other lung disorders has also been investigated. Hypoxia causes an elevation of proinflammatory mediator expression and the presence of alternatively activated macrophages during the progression to hypoxic pulmonary hypertension [114]. Hypoxia evokes the release of a large number of lung epithelial cell-derived exosomes following endoplasmic reticulum (ER) stress. These exosomes are enriched for caspase-3 and mediate the activation of alveolar macrophages and the initiation and propagation of the inflammatory responses resulting in lung injury. These results highlight the role of exosomes in lung epithelial cell-macrophage communication in the development of tissue injury [115].

There are few therapeutic options for Idiopathic Pulmonary Fibrosis (IPF) and it has a dismal median survival of 2-3 years [116]. It has recently been reported that exosomal miRNAs in IPF patients contain decreased levels of antifibrotic miRNAs such as miR-141 and increased levels of fibrogenic miRNAs such as miR-7 compared with control subjects. There was a good correlation between the degree of miR-7 upregulation and the burden of disease and also between miR-125b upregulation and milder disease. These findings demonstrated the role of exosomal miRNAs as the biomarkers to gain an insight into the pathogenesis of IPF [117].

Cystic fibrosis (CF) is a genetic lung disorder, characterized by a deficiency in chloride channel activity, the CF transmembrane conductance regulator (CFTR), resulting in the massive neutrophil granulocyte influx in the airways and mucostasis [118]. Early evidence suggested that bronchial epithelial cells from CF patients demonstrate enhanced release of exosomes [119]. These findings provided an opportunity to use biofluid exosomes as a rich and noninvasive source of diagnosis biomarkers in CF patients.

The enzyme prolyl endopeptidase (PE) is essential for the production of the neutrophil chemoattractant tripeptide Pro-Gly-Pro (PGP) from collagen and plays a role in airway remodelling and inflammation [120]. PE is released from airway epithelial cells via exosomes and this release can be enhanced by the bacterial mimic LPS acting through TLR4. Sputum samples from CF patients who have persistent bacterial infection demonstrate the presence of exosomes with increased PE levels which may be important for the hyperinflammatory state observed in these patients [120]. In gut biology, basolateral release of exosomes from epithelial cells is linked to antigen presentation, whereas luminal release of exosomes occurs in response to TLR4 activation [121]. Analysis of this differential release of exosomes should be undertaken in CF cells.

Evidence suggests that airway epithelial cells release vesicles with distinct physical properties and sizes dependent upon their origin and that this may be particularly important in disease states [122]. For example, the type and amount of mucin on the exosomes surface define their size and charge and this will alter in CF patients which are characterized by excessive mucus production. In a recent abstract, the same group reported that there were changes in the exosomes miRNA and protein cargo that reflected the cellular changes occurring after an inflammatory challenge; this study shows that exosomes can change the airway microenvironment to a hyperinflammatory state in lung CF [123].

Restoration of CFTR function is a major goal of CF research and recent evidence suggests that utilization of exosomes may be of benefit in the treatment of this disease [118]. Exosomes derived from CFTR-positive Calu-3 cells or from A549 cells transduced with an adenoviral vector overexpressing a GFP-tagged CFTR (GFP-CFTR) were able to deliver the GFP-CFTR glycoprotein and mRNA (GFP-CFTR) to CFTR-deficient nasal epithelial cells and restore CFTR function in a dose-dependent manner [118].

Overall, the above studies emphasize the key roles of exosomes in cell-to-cell communication within the lung microenvironment and also in pathogenicity. Not only are exosomes important as diagnostic biomarkers but also they offer new potential avenues in the lung diseases treatment. In the next section, we review the therapeutic roles of exosomes in respiratory disorders.

## 12. Exosomes and Therapeutic Roles in Lung Diseases: Clinical Trials and Future Perspectives

Exosomes are involved in intercellular communication and the maintenance of homeostatic functions in the lung microenvironment. Lung exosomes originate from a broad range of respiratory cell types including structural and immune cells. These exosomes appear to be expressed in different numbers dependent upon disease status and have distinct disease-specific constituents. Based on the present evidence, exosomes may provide novel diagnostic biomarkers for a broad range of pulmonary diseases and may also be used for therapeutic interventions.

Exosomes derived from mesenchymal stem cells (MSC) have recently been proposed as having the potential for tissue repair, wound healing, and lung tissue regeneration [119]. These MSC-derived exosomes have special properties including antiapoptotic and anti-inflammatory actions [124] and MSC-based therapy for the treatment of acute respiratory distress syndrome (ARDS) is currently in phase 1 clinical trials [125]. MSC-derived exosomes are beneficial in animal models of ARDS, where they induce the expression of keratinocyte growth factor (KGF) in the injured alveolus which restores lung protein permeability and reduces lung inflammation in the mouse lung [126]. Immunotherapy using tumor antigen-loaded DC-derived exosomes (dexosomes) is in phase I clinical trials for NSCLC [127]. This trial demonstrated that dexosome therapy was feasible and safe and caused the induction of both innate and adaptive immune responses in these patients. Further studies are required to examine the effect on disease stability and on long-term survival.

## 13. Conclusion

Exosomes mediate cell-cell communication and have several advantages for the delivery of information to target cells. Exosomes have been identified in body fluids where they are stable and can transfer information/mediators between organs irrespective of distance. Exosomes are heterogeneous in size and content and these alter with disease status making them potentially useful as diagnostic biomarkers for disease or drug efficacy. Exosomes can also deliver specific mRNAs, miRNAs, and proteins directly to recipient cells and alter their function, which suggests that they may be an effective tool to target therapy to specific cells and organs.
